# Carbon dots-fed *Shewanella oneidensis* MR-1 for bioelectricity enhancement

**DOI:** 10.1038/s41467-020-14866-0

**Published:** 2020-03-13

**Authors:** Chenhui Yang, Hüsnü Aslan, Peng Zhang, Shoujun Zhu, Yong Xiao, Lixiang Chen, Nasar Khan, Thomas Boesen, Yuanlin Wang, Yang Liu, Lei Wang, Ye Sun, Yujie Feng, Flemming Besenbacher, Feng Zhao, Miao Yu

**Affiliations:** 1 State Key Laboratory of Urban Water Resource and Environment, School of Chemistry and Chemical Engineering, Harbin Institute of Technology, 150001 Harbin, China; 2 Condensed Matter Science and Technology Institute, School of Instrumentation Science and Engineering, Harbin Institute of Technology, 150001 Harbin, China; 3 iNANO Centre, Aarhus University, 8000 Aarhus, Denmark; 4 Sino-Danish Centre for Research and Education (SDC), 8000 Aarhus, Denmark; 5 State Key Laboratory of Urban Water Resource and Environment, School of Environment, Harbin Institute of Technology, 150090 Harbin, China; 6 Laboratory of Molecular Imaging and Nanomedicine, National Institute of Biomedical Imaging and Bioengineering (NIBIB), National Institutes of Health (NIH), Bethesda, MD 20892 USA; 7 CAS Key Laboratory of Urban Pollutant Conversion, Institute of Urban Environment, Chinese Academy of Sciences, 361021 Xiamen, China; 8 Center for Electromicrobiology, Aarhus University, 8000 Aarhus, Denmark

**Keywords:** Biotechnology, Environmental sciences, Energy science and technology, Materials science, Nanoscience and technology

## Abstract

Bioelectricity generation, by *Shewanella oneidensis* (*S. oneidensis*) MR-1, has become particularly alluring, thanks to its extraordinary prospects for energy production, pollution treatment, and biosynthesis. Attempts to improve its technological output by modification of *S. oneidensis* MR-1 remains complicated, expensive and inefficient. Herein, we report on the augmentation of *S. oneidensis* MR-1 with carbon dots (CDs). The CDs-fed cells show accelerated extracellular electron transfer and metabolic rate, with increased intracellular charge, higher adenosine triphosphate level, quicker substrate consumption and more abundant extracellular secretion. Meanwhile, the CDs promote cellular adhesion, electronegativity, and biofilm formation. In bioelectrical systems the CDs-fed cells increase the maximum current value, 7.34 fold, and power output, 6.46 fold. The enhancement efficacy is found to be strongly dependent on the surface charge of the CDs. This work demonstrates a simple, cost-effective and efficient route to improve bioelectricity generation of *S. oneidensis* MR-1, holding promise in all relevant technologies.

## Abstract (web summary)

Bacterial fuel cells have generated attention with the prospect of green energy production; current research is focused on optimising the system to improve efficiency. Here, the authors report on the feeding of carbon dots to S. oneidensis MR-1 to enhance metabolic activity and bioelectric generation.

## Introduction

Bioelectricity produced by electrochemically active bacteria has sparked substantial interests, showing considerable prospects for sustainable energy production, environmental pollution treatment, as well as fabrication of metal nanoparticles^1–3^. Among various bacteria applied for this purpose, *Shewanella oneidensis* (*S. oneidensis*) MR-1 is the most representative, due to their prominent competency on electron generation as well as electron transfer from the quinone and quinol pool in the cytoplasmic membrane to extracellular electron acceptors, e.g., minerals containing Fe(III) or electrodes of fuel cells^4,5^.

Modification of *S. oneidensis* MR-1 is one of the primary strategies to improve their performance for bioelectricity generation. Several successful means have been proposed, including genetic engineering to provide purposeful protein expression^6^, biologically generated nanoparticles (e.g., Au, Pd) on the surface or inside the bacteria for direct electron-transfer enhancement^3,7^, and synthetically conjugated oligoelectrolytes through bacterial outer membrane intercalation for transmembrane electron-transfer enhancement^8^. However, the sophistication and high cost of these methods, as well as the relatively low enhancement, have been recognized as a bottleneck in real-life applications^9^. Therefore, efficient enhancement of *S. oneidensis* MR-1’s capability for bioelectricity generation based on facile and cost-effective methods would be highly desirable.

Recently, carbon dots (CDs) have become particularly attractive, due to their unique electronic and physicochemical properties, such as high electron-transfer efficiency, excellent stability, reliable biocompatibility, and distinct optical absorption and photoluminescence (PL)^10^. So far, CDs have demonstrated promising performance across a large variety of technical practices, e.g., light emission, photo/electro-catalysis, sensing of heavy metal ions, and biological applications of in vivo imaging and antitumor treatment^11–18^. However, their interaction with bacteria, especially the potential in bioelectrical systems, remains unexplored.

**Fig. 1:**
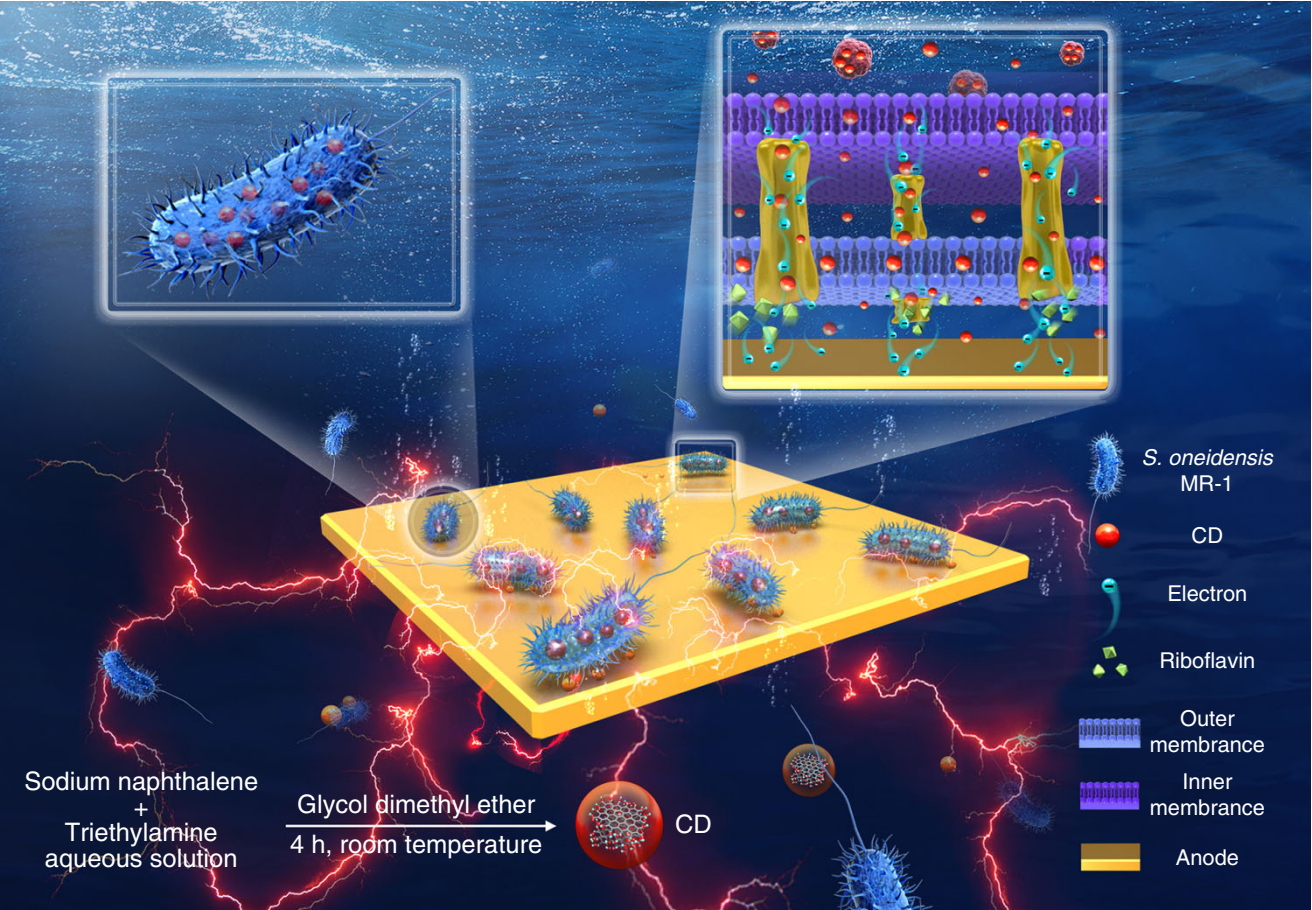
Illustration of the synthesis of the CDs and the CDs-fed *S. oneidensis* MR-1 for enhanced bioelectricity generation.

Herein, we report on the application og CDs to boost the bioelectricity generation of *S. oneidensis* MR-1. The water-soluble CDs employed in this work are synthesized by sodium naphthalene reagent and triethylamine at room temperature (Fig. 1). The CDs are found to be efficiently up-taken by *S. oneidensis* MR-1 and to accelerate bacterial metabolism, showing increased intracellular charge, adenosine triphosphate (ATP) level, substrate consumption, extracellular secretion, as well as transmembrane, and extracellular electron transfer. The addition of the CDs can also promote cellular adhesion, electronegativity, and biofilm formation. A substantial boost of bioelectricity generation by the CDs-fed *S. oneidensis* MR-1 is demonstrated in both three-electrode microbial electrochemical cells (MECs) and microbial fuel cells (MFCs). The maximum current value, total charge, maximum cell voltage, and power output are increased ∼7.34, 5.63, 3.78, and 6.46 fold, respectively, compared with the control group without the CDs. The efficacy of bioelectricity enhancement is found to be strongly correlated to the surface charge of the CDs. This first application of CDs to *S. oneidensis* MR-1 introduces a simple, efficient route to improve electron generation and the subsequent electron transfer, holding considerable potential in bioelectricity generation and bacterial-related redox reactions.

## Results

### Characterization of the CDs

**Fig. 2:**
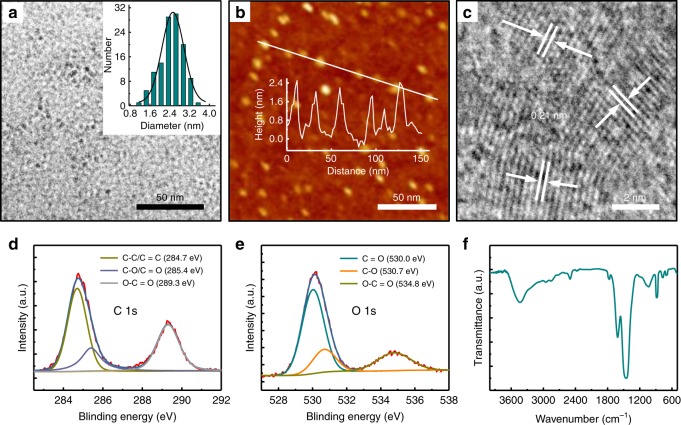
Morphology and composition of the CDs. **a** TEM image and size distribution. **b** AFM image and height profile of the linescan. **c** HRTEM image of the CDs. **d** C 1*s* XP, **e** O 1*s* XP, and **f** FTIR spectra of the CDs.

Transmission electron microscope (TEM) micrographs (Fig. 2a) and atomic force microscope (AFM) images (Fig. 2b) revealed quasi-spherical morphology of the CDs with an average diameter of ∼2.5 nm and a height of ∼2.2 nm. The high-resolution (HR) TEM micrograph (Fig. 2c) showed evident crystalline structure, with a lattice distance of ∼0.21 nm corresponding to the in-plane spacing of graphene (100)^17,18^. The chemical composition of the CDs was analyzed by X-ray photoelectron spectroscopy (XPS) and Fourier transform infrared (FTIR) spectroscopy. The XPS survey (Supplementary Fig. 1) presented two peaks corresponding to C and O, respectively. C 1*s* XP spectrum (Fig. 2d) showed binding energies of 284.7, 285.4, and 289.3 eV for three components, i.e., C=C/C–C, C–O/C=O, and O–C=O, respectively. O 1*s* XP spectrum (Fig. 2e) showed three sub-peaks at 530.0, 530.7, and 534.8 eV, which can be assigned to C=O, C–OH, and O–C=O. Consistently, FTIR analysis (Fig. 2f) showed characteristic peaks at 3433 cm^−1^ corresponding to C–OH stretching vibration, those at 2953 and 2860 cm^−1^ corresponding to the C–H stretching vibration, those at 1769 and 1614 cm^−1^ corresponding to C = O stretching and C=C stretching vibration, those at 1452 cm^−1^ corresponding to C–H bending vibration, and those at 1080–1020 cm^−1^ corresponding to C–O–C bending vibration. All these results indicate that the CDs are rich in phenolic hydroxyl group. Such functional group introduces ample electrons on the CDs’ surface, as confirmed by the zeta potential measurement (−41.3 ± 1.5 mV).

### The biocompatibility of the CDs with *S. oneidensis* MR-1

**Fig. 3:**
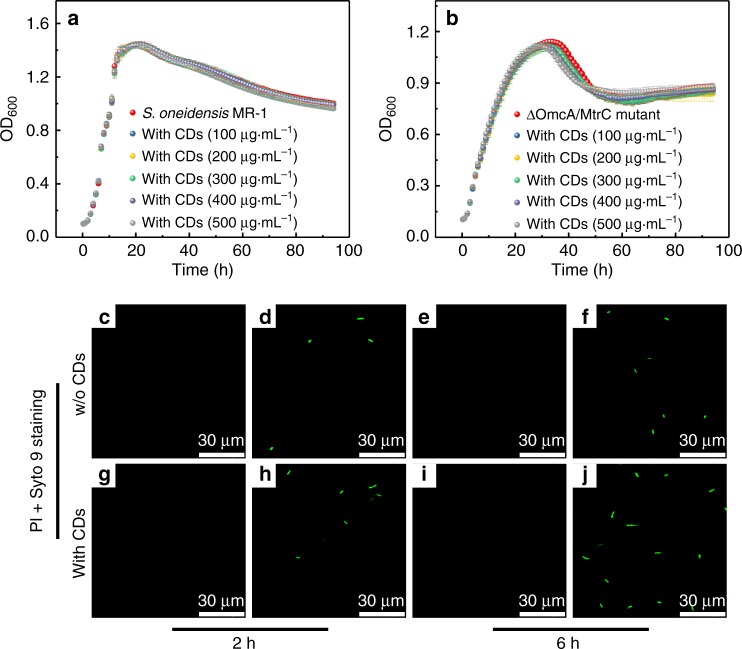
The viability of the CDs-fed *S. oneidensis* MR-1. LB growth curves of **a** Wild type *S. oneidensis* MR-1 and **b** ΔOmcA/MtrC mutant after incubation with the CDs at various concentrations (100–500 µg mL^−1^). Data in **a** and **b** are expressed as the mean ± standard deviation. Fluorescence imaging of Wild Type *S. oneidensis* MR-1 **c**–**f** without and **g**–**j** with the CDs’ addition for 2/6 h, stained by PI and Syto 9, where no red fluorescence from PI is in presence, indicating the good integrity of the cellular membrane. The excitation and emission wavelengths are 535 and 617 nm for PI, 485 and 498 nm for Syto 9.

Luria-Bertani (LB) growth curves were first measured to assess the viability of Wild Type and ΔOmcA/MtrC mutant *S. oneidensis* MR-1 incubated with the CDs at different concentrations (Fig. 3a, b). After incubation with the CDs up to 500 µg mL^−1^ (the working concentration of the CDs in the MEC and MFC systems addressed below was as low as 100 µg mL^−1^) for 94 h, the cell viability of both types of bacteria was found to be maintained above 96%. Moreover, fluorescence staining using propidium iodide (PI) and Syto 9 was applied to explore the integrity of the cellular membrane (Protocol refers to Supplementary Fig. 2). While PI (red fluorescence) penetrates into cells with damaged membranes exclusively and indicates the dead cells, Syto 9 (green signal) can stain both living and dead cells. *S. oneidensis* MR-1 cells treated with 70% ethanol were used as the control group for dead cells (Supplementary Figs. 3, 4). For all the cells treated with the CDs for 2 or 6 h, green fluorescence was exclusively distinguished like the case of pristine cells (Fig. 3c−j). All these results confirm the high compatibility and low cytotoxicity of the CDs for *S. oneidensis* MR-1.

### Uptake of the CDs by *S. oneidensis* MR-1

**Fig. 4:**
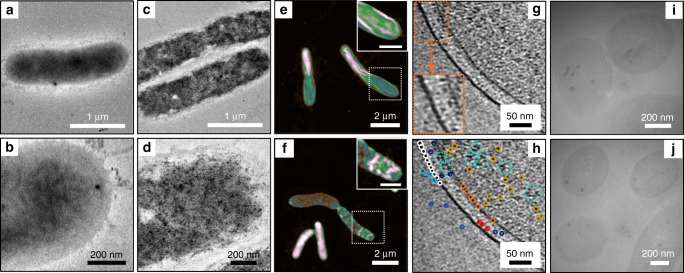
Cellular uptake of the CDs by *S. oneidensis* MR-1. TEM images of *S. oneidensis* MR-1 cells **a**, **b** before and **c**, **d** after 6 h incubation with the CDs. **e**, **f** AFM images of dehydrated pristine and CDs-fed *S. oneidensis* MR-1, depicting altered roughness in the presence of CDs. Scale bars in the inserts are 1 μm. **g**, **h** TEM image of a CDs-fed cell constructed using cryo-electron tomography, where the LBDs located at the different zones of the cell are marked by the solid-line circles in different colors. **i**, **j** TEM images of cross-section slices of the CDs-fed cells, where the dark clusters are assigned to accumulated LBDs.

The TEM images of pristine *S. oneidensis* MR-1 cells showed a relatively smooth profile and uniform contrast (Fig. 4a, b), whilst obvious black spots were found from the images of the cells after 6 h incubation with the CDs (Fig. 4c, d). AFM images (Fig. 4e, f) indicated that the roughness of the dehydrated CDs-fed cells was ∼49% higher than that of the pristine cells (104.3 ± 28.1 nm vs. 70.0 ± 22.1 nm, *n* = 24). According to the literature^19,20^, in addition to the attachment on the cell surface, the profile of intracellular particles or their clusters (with size detectable by AFM) can be reflected from the varied roughness of dehydrated cells, as the cells have lost the flexible and malleable cytoskeleton. To further explore whether the CDs were attached on the cell surface specifically or also being able to distribute inside the cells, cryo-electron tomography was employed. A pronounced difference in contrast was observed in the tomograms between the membranes of the CDs-fed cells (Fig. 4g, h) and those of pristine cells (Supplementary Fig. 5a), where the former appeared much darker with aligned blacked dots within the membranes (pointed by the white and light green circles), whilst the latter showed relatively lighter and uniform contrast. It is possible that the CDs interacting intimately with the cell membrane can change the density, charge and/or structure of the membrane, inducing the observed difference in electron scattering. Moreover, besides the small dark spots (typical size ≤3.6 nm, Supplementary Fig. 5b) corresponding to the inherent components observed in the pristine cells, abundant additional larger black dots (LBDs) with an average size of ∼5.4 nm (Supplementary Fig. 5c) were observed everywhere in the cells, including the cytoplasm, periplasm, inner membrane, and outer membrane in the CDs-fed cell. These LBDs are very likely attributed to the assemblies of CDs with the cellular contents. Although the average size of dry CDs was ∼2.5 nm (Fig. 2a), a significantly enlarged size in the cryo-electron image is sensible considering the solution environment and the coordination of CDs with the cytochromes and/or other components inside the cells. It is noticed that the LBDs were able to accumulate into small patches in the cytoplasm, as marked by the dashed circles. TEM imaging of the cross-section cell slices (Fig. 4i, j and Supplementary Fig. 6) supported this observation by revealing the dark clusters, with a typical size of 20‒50 nm in the cytoplasm and periplasm of the CDs-fed cells. Since the sample thickness of the cross-section cell slice was much larger than the scanning range of the cryo-electron tomography, the contrast of the clusters was more significant. All of these results suggest the efficient cellular uptake of the CDs by *S. oneidensis* MR-1 cells.

### Adhesion and electronegativity of the CDs-fed cells

**Fig. 5:**
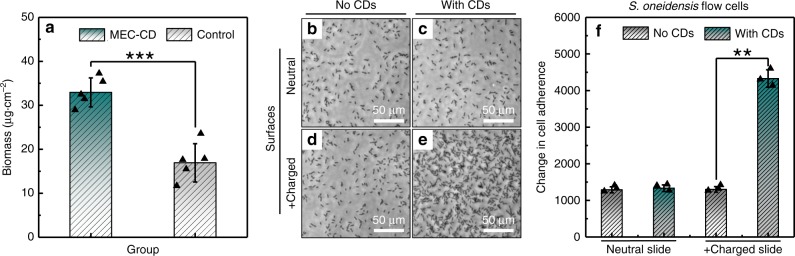
Increased adhesion of the CDs-fed *S. oneidensis* MR-1. **a** Increased biomass on the MEC anode by addition of the CDs (*n* = 5), where *MEC-CD* and control represent the groups of *S. oneidensis* MR-1 with and without the CDs, respectively. Optical images of *S. oneidensis* MR-1 cells **b**, **d** without and **c**, **e** with the CDs on the neutral (**b**, **c**) and positively charged (**d**, **e**, +charged) glass surfaces, respectively. **f** Surface coverage of *S. oneidensis* MR-1 with/without the CDs on different slides (*n* = 3). Data in **a** and **f** are expressed as the mean ± standard deviation. Two-tailed Student’s *t*-test: *p < 0.05, **p < 0.01, and ***p < 0.001.

For electricity generation in the bioelectrical systems of bacteria, the cellular adhesion and the cell surface electronegativity play a vital role in determining the junctions of the cells with the anode and the direct extracellular electron transfer rate. To dig out these properties of the CDs-fed *S. oneidensis* MR-1, the growth of *S. oneidensis* MR-1 on MEC anodes (carbon cloth) with/without the CDs was first analyzed in static culture (Fig. 5a). The cell population with the CDs were found to be evidently higher (32.94 ± 3.30 μg cm^−2^ vs. 16.92 ± 4.35 μg cm^−2^). We then conducted flow-cell experiments to further explore the cellular adhesion and accumulation in the dynamic case. Briefly, phosphate buffer saline (PBS) containing sodium lactate and *S. oneidensis* MR-1 from the same culture with/without the CDs were flown on (1) neutral glass slides, and (2) positively charged glass slides for 6 h (Supplementary Fig. 7). Optical microscopy images showed that there was no significant difference of adhesion on the non-charged surface between the pristine cells and the CDs-fed ones (Fig. 5b, c). However, on positively charged slides, a drastically increased cell adherence hence higher surface coverage was observed in the presence of CDs (Fig. 5d−f). This combined view of results from the biomass and flow-cell experiments suggests the incremental cellular electronegativity by the CDs, consistent with the densely aligned CDs in the cell membrane revealed in the TEM images (Fig. 4g, h).

### Metabolism enhancement by the CDs’ addition

Remarkably, the CDs accelerate the metabolic rate of *S. oneidensis* MR-1. The metabolism in this context refers to intracellular charge production, ATP level, extracellular secretion, and transmembrane and extracellular electron transfer.

**Fig. 6:**
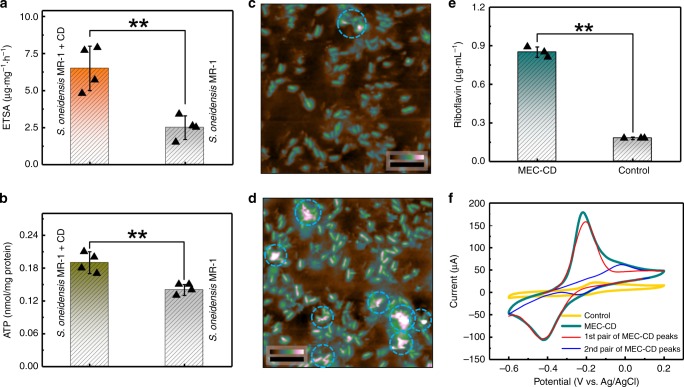
Increase of metabolic rate of the CDs-fed *S. oneidensis* MR-1. **a** ETSA measurement of *S. oneidensis* MR-1 with/without the CDs addition for 12 h incubation (*n* = 4). **b** ATP level of the pristine cells and cells incubated with the CDs for 12 h (*n* = 4). **c**, **d** AFM results of living *S. oneidensis* MR-1 on positively charged glass slides without the CDs and after 6 h-incubation with the CDs. The color bars indicate height difference from 0 to 900 nm and the scale bars are 10 µm. **e** Riboflavin concentration (*n* = 3) and **f** CV curves (10 mV s^**‒**1^) of *MEC-CD* and control group, where *MEC-CD* and Control group represent the MEC of *S. oneidensis* MR-1 with and without CDs’ addition, respectively. Data in **a**, **b**, **e** are expressed as the mean ± standard deviation. Two-tailed Student’s *t*-test: *p < 0.05, and **p < 0.01.

Intracellular charge generation level of the CDs-fed *S. oneidensis* MR-1 was examined by using electron transport system activity (ETSA) assay^21^. Briefly, the cells were incubated in MEC for 12 h, then added into 2,3,5-triphenyltetrazolium chloride solution at 37 °C for 30 min. As shown in Fig. 6a, the ETSA value of the CDs-fed cells was evidently higher than that of the pristine ones (6.5 ± 1.5 μg mg^−1^ h^−1^ vs. 2.5 ± 0.8 μg mg^−1^ h^−1^), whilst the CDs alone did not contribute to the ETSA value.

As ATP represents the energy stored from the intracellular redox reactions of bacteria^22^, the metabolic activity of the cells with/without the CDs’ addition was further evaluated by an ATP assay (Fig. 6b). Consistently, the ATP level of the CDs-fed cells increased to ∼136% as that of the pristine cells (0.19 ± 0.02 nmol/mg protein vs. 0.14 ± 0.01 nmol/mg protein), while the influence of the CDs alone (without the cells) on the ATP measurement was negligible.

In the absence of CDs, extracellular secretion was very limited (Fig. 6c). After 6 h incubation with the CDs, extracellular secretion layer was abundantly growing into high-coverage continuous domains (Fig. 6d, as circled by the blue dashed lines), forming the biofilm. To quantitatively examine the extracellular secretion, we then measured the concentration of riboflavin, an important redox-active compound secreted by *S. oneidensis* MR-1 as a result of bacterial metabolic activities^23^, using high performance liquid chromatography (HPLC, Fig. 6e). The concentration of riboflavin from the CDs-fed bacteria was largely increased (0.85 ± 0.04 μg mL^−1^ vs. 0.18 ± 0.01 μg mL^−1^), which further confirmed the enhanced extracellular secretion by the CDs’ addition.

The transmembrane electron transfer refers to delivering electrons from redox carriers in the cytoplasm to the cell outer surface over the cell membranes and periplasm. The extracellular electron transfer activity is mainly divided into two mechanisms according to the transfer path: (1) direct electron transfer from the cells to extracellular electron acceptors via bacterial membrane-bounded cytochromes and/or conductive bacterial pili “nanowires”^24–26^; (2) mediated electron transfer via soluble redox mediators, e.g., flavins^23,27,28^. Incorporating with the CDs was found to promote the electrochemical behavior of *S. oneidensis* MR-1. Cyclic voltammetry (CV) curves in the MEC system of the CDs-fed cells (denoted as *MEC-CD*) were measured. The redox process was confirmed to originate from the bacteria since no electrochemical activity from carbon cloth (as the anode material) with the CDs was observed (Supplementary Fig. 8). Cyclical scans in forward and reverse directions within the electrical potential range of −0.60 to 0.20 V (vs. Ag/AgCl electrode) were recorded after the substrate compound (lactate) was exhausted (Fig. 6f). Unlike the control group that only showed weak redox peaks, two pairs of pronounced redox peaks were observed in *MEC-CD*: (1) the 1st pair, i.e., the reduction peak current of −103.5 μA at −0.43 V and oxidation peak current of 157.8 μA at −0.20 V, where the enhancement is correlated with the mediated extracellular electron transfer increased by additional riboflavin excretion^23^ upon the CDs’ addition; and (2) the 2nd pair, i.e., the reduction peak current of −4.8 μA at −0.24 V and oxidation peak current of 61.9 μA at −0.02 V, where the enhancement is associated with the improved transmembrane electron transfer^29,30^.

The increased intracellular electron generation, higher ATP level, more vigorous extracellular secretion, and enhanced transmembrane and extracellular electron transfer all emphasize the positive and effective contribution of the CDs to the elevated metabolism of *S. oneidensis* MR-1.

### Interaction of the CDs with cytochromes

**Fig. 7:**
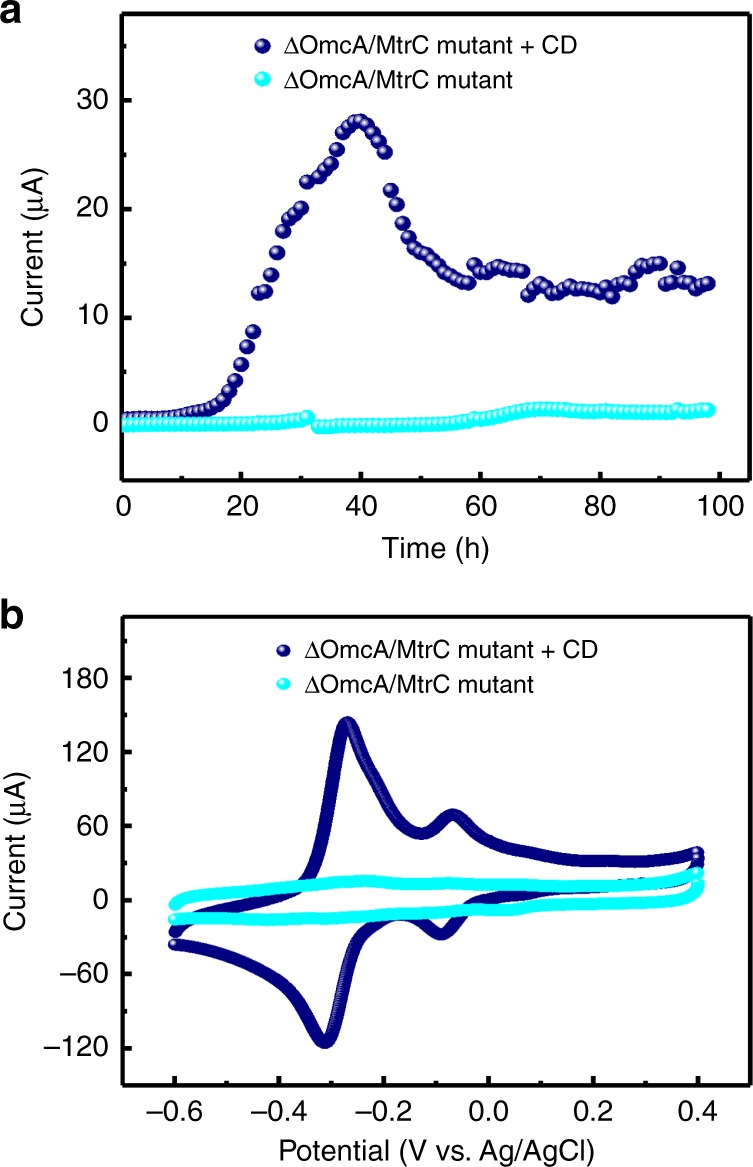
Increased charge transfer of ΔOmcA/MtrC mutant by incorporating with the CDs. **a** Current output and **b** CV curves (10 mV s^−1^) of ΔOmcA/MtrC mutant with/without the addition of the CDs, showing that the participation of the CDs can efficiently improve the electrochemical performance of ΔOmcA/MtrC mutant cells.

*C*-type cytochromes are a family of iron-containing proteins (e.g., MtrA, MtrB, MtrC, OmcA, and CymA) in *S. oneidensis* MR-1, which work synergistically to enable electron transfer throughout the cell membrane^31^. The boosted transmembrane electron transfer (Fig. 6f) together with the participation and increased size of CDs in the cell membrane and periplasm (Fig. 4g, h) of the CDs-fed *S. oneidensis* MR-1 thus suggests the possible interaction between the CDs and these cytochromes. To further explore this possibility, we measured the current output of mutated *S. oneidensis* MR-1 lacking OmcA and MtrC of the outer membrane (ΔOmcA/MtrC mutant) with/without the CDs (Fig. 7a). For the group of ΔOmcA/MtrC mutant alone, the peak current output was rather weak (as low as ∼ 1.5 μA after 70 h), due to the fractured electron transfer chain^31^. As a sharp contrast, the output from the group of ΔOmcA/MtrC mutant with the CDs reached ∼28.1 μA within 40 h. Moreover, distinctly different from the tiny redox peaks of the control group, both pairs of the redox peaks were significant for the CDs-fed ΔOmcA/MtrC mutant (Fig. 7b). These results suggest that the CDs can not only accelerate the electron transfer of normal wide *S. oneidensis* MR-1 but also interact with ΔOmcA/MtrC mutant to restore its compromised electron transfer capability.

In addition, fluorescence imaging was employed to demonstrate CDs-cytochromes interaction intuitively. The CDs possessed pronounced PL emission, with in vitro imaging potential (Supplementary Figs. 9–10). Nevertheless, not only the pristine cells but also the CDs-fed *S. oneidensis* MR-1 and CDs-fed ΔOmcA/MtrC mutant showed no fluorescence signal (Supplementary Fig. 11). Given the significant cellular uptake, this typical PL quenching effect thus suggests the binding and charge transfer of the CDs with the intracellular components. It has been well addressed in the literature that, the phenolic hydroxyl group of the CDs enables electrostatic and/or coordination interaction with cytochromes or enzymes^32^. Considering the wide distribution of the CDs in the cells, it is rational to believe that the enhanced charge transfer and metabolic rate of the CDs-fed cells are associated with the interaction between the CDs and various cytochromes/enzymes of *S. oneidensis* MR-1.

### Bioelectricity generation in MEC and MFC

**Fig. 8:**
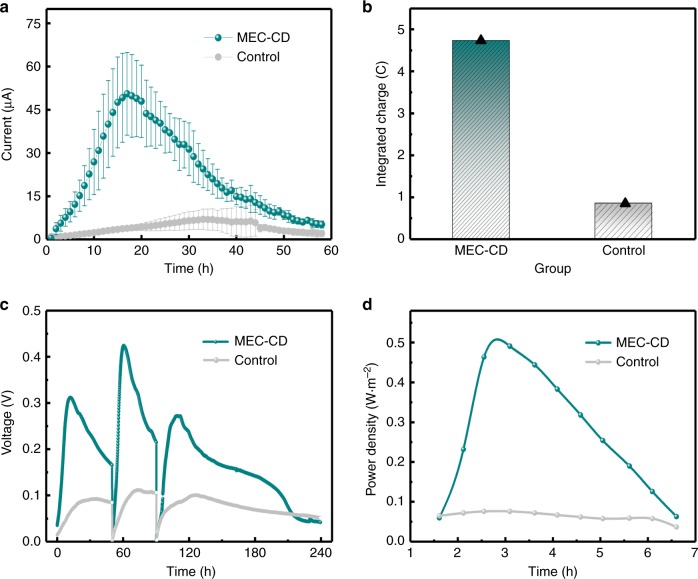
Bioelectricity generated from the MEC and MFC systems. **a** Current output of *MEC-CD* and control group (*n* = 3), where *MEC-CD* and Control represent the groups of *S. oneidensis* MR-1 with and without the CDs, respectively. Data are expressed as the mean ± standard deviation. **b** Integrated charge of *MEC-CD* and Control group. **c** Time course vs. cell voltage for three cycles and **d**, power output of the *MFC-CD* and the control, where *MFC-CD* and control represent the MFC of CDs-fed and pristince *S. oneidensis* MR-1.

The effect of the CDs on the enhancement of bioelectricity generation was investigated in both the MEC and MFC systems. The MEC measurements were carried out in a single-chamber electrochemical reactor (42 mL) using a three-electrode system with a potential of 0.2 V applied at the anode (vs. Ag/AgCl electrode). As depicted in Fig. 8a, for the control group, i.e., pristine *S. oneidensis* MR-1, the current increased gradually and reached the maximum point (6.89 ± 3.64 μA) at 33 h; while with the CDs (100 μg mL^‒1^), the current of the *MEC-CD* increased much quicker and accomplished the maximum current at 17 h, which is 50.54 ± 14.38 μA (*n* = 3) and 7.34 times as that of the control one. It is noticed that the current output of the *MEC-CD* dropped much earlier than that of the Control group, implying quicker consumption hence earlier reduced concentration of the electron donor^33^, i.e., lactate in the present case. The results further support the elevated metabolic level of the CDs-fed *S. oneidensis* MR-1. The integrated total charge collected by the *MEC-CD* was ∼4.73 C, 5.63 times as that of pristince cells (∼0.84 C) (Fig. 8b).

For the MFC measurements, a double-chamber reactor (100 mL of each chamber) was used at a constant resistant load of 1.0 kΩ. The maximum cell voltage of the *MFC-CD* was ∼0.42 V, much higher than that of the Control group (∼ 0.11 V) (Fig. 8c). For the cell voltage ranging from 0.55 to 0.05 V, the current density of the *MFC-CD* was significantly improved (Supplementary Fig. 12). For instance, at 3 h, the current density for the *MFC-CD* and control was 1.23 and 0.19 A m^−2^, respectively. Meanwhile, the maximum power density (Fig. 8d) of the *MFC-CD* was 0.491 W m^−2^, 6.46 folds as that of the Control (0.076 W m^−2^). All these results directly demonstrate that the addition of CDs can effectively enhance the electricity generation and power output.

### Influence of surface charges of the CDs

**Fig. 9:**
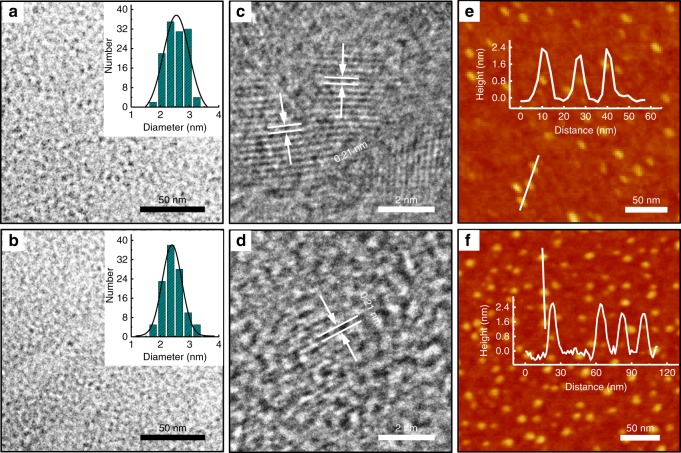
Morphology of CD-X1 and CD-X2. **a**, **b** TEM images and size distribution, **c**, **d** HRTEM images, and **e**, **f** AFM images and height profile of the linescan of CD-X1and CD-X2.

To explore the CDs’ surface charge on the efficacy of bioelectricity enhancement, two additional samples, i.e., CD-X1 and CD-X2, were synthesized, with ethylenediamine/propylamine as the precursor instead of triethylamine, respectively. Seen from the TEM (Fig. 9a–d) and AFM results (Fig. 9e, f), the CD-X1 and CD-X2 also appeared as quasi-spherical particles, with an average diameter of ∼2.6 and 2.4 nm, lattice spacing of ∼0.21 nm, and height of ∼2.2 and 2.3 nm, respectively. Albeit the similar morphology and structure, XPS analysis (Supplementary Fig. 13 and Supplementary Table 1) indicated that the CD-X1 and CD-X2 had a lower content of −O−C=O and C−O/C=O (Supplementary Fig. 14), compared with the CD. Consistently, surface zeta potential measurements (Supplementary Fig. 15) further confirmed that |*ζ*_CD_ | (−41.3 ± 1.5 mV) > |*ζ*_CD-X1_ | (−33.7 ± 1.7 mV) > |*ζ*_CD-X2_ | (−22.6 ± 1.2 mV), indicating their different surface charge.

The CD-X1 and CD-X2 were then applied in the MEC system of *S. oneidensis* MR-1. It was found that the maximum current generated by *MEC-CD-X1* and *MEC-CD-X2* (100 μg mL^−1^) (Supplementary Fig. 16a) were 34.41 ± 4.52 and 20.67 ± 5.35 μA (*n* = 3), also much higher than that of the control group (6.89 ± 3.64 μA, *n* = 3). Moreover, increased riboflavin, biomass on the anode, and redox current were also observed from the *MEC-CD-X1* and *MEC-CD-X2* (Supplementary Fig. 16c–e). However, the capability of the three CDs specimens to induce these enhancements was distinct from one another. In terms of efficacy, the CD was better than the CD-X1, which was better than the CD-X2. We then conclude that more abundant surface electrons of the CDs can benefit their performance on bioelectricity generation enhancement.

## Discussion

Except for the modification of the anodes^34,35^, the major efforts to enhance the bioelectricity produced by *S. oneidensis* MR-1 in the literature have been focused on improving extracellular electron transfer^5–9^. However, rather less attention was paid on the variation of the bacteria. In this work, feeding with the CDs, *S. oneidensis* MR-1 showed an accelerated metabolic rate, as supported by the evidence of (1) more intracellular electrons, (2) higher ATP level, (3) quicker substrate utilization, (4) incremental extracellular excretion and accumulated biofilms, and (5) boosted transmembrane and extracellular electron transfer. These increased metabolic activities are benefited from the efficient cellular uptake/attachment of the CDs and their wide distribution in the cells. In this case, the interaction of CDs with the cytochromes/enzymes in the cell may modulate the intracellular redox reactions hence the metabolic level; meanwhile, the transmembrane and extracellular electron transfer are expedited, which are known for promoting metabolic activities in turn^21,36,37^, e.g., increasing intracellular electron and energy, excretion of flavins, etc.

The contributions of the CDs to transmembrane and extracellular electron transfer are summarized as follows. (1) The surface oxygen related groups of CDs increase the electronegativity of the bacteria hence improve their static and dynamic adhesion and charge transfer at the anode. (2) The transport of electrons from the cytoplasmic membrane across the periplasm and outer membrane to the extracellular space strongly relies on an efficient path of cytochromes chains^31,38^. The interaction of the CDs with the cell content and the densely aligned CDs in the outer membrane possibly benefit the electron transfer network in two ways, i.e., increasing the packing density of transfer path and improving its electrical conductivity, as the CDs’ carbon core is known for high conductivity^14,39^. (3) The CDs can even restore charge transfer capability of ΔOmcA/MtrC mutant that suffers from compromised electron-transfer path. (4) The accelerated metabolic rate by the CDs may further urge the electron transport. (5) The incremental flavin-based molecules largely elevate the indirect extracellular electron transfer. (6) Considering biofilms as complex 3D networks, one can also argue that the CDs may also benefit intercellular signaling and charge transfer by increasing extracellular secretion and biofilm formation^40,41^.

To conclude, we have successfully developed a facile and cost-effective strategy to enhance bioelectricity generation by feeding the CDs to *S. oneidensis* MR-1. The CDs can be efficiently uptaken by and interact with *S. oneidensis* MR-1, facilitate the transmembrane and extracellular electron transfer, elevate the cell metabolic rate, and increase cellular adhesion and electronegativity. Both the greatly enhanced current output in MECs and the power output in MFCs show notable improvement in bioelectricity production.

This study’s importance is to provide a proof of concept for CDs-facilitated enhancement of bioelectricity generation. *S. oneidensis* MR-1 is widely utilized for bioenergy applications, wastewater treatment^1,2,42^, and biofabrication of metal nanomaterials^3,7,43,44^. The promotion of metabolic activities and electron generation/transfer of *S. oneidensis* MR-1 by CDs’ addition holds tremendous potential to improve, diversify, and innovate the aforementioned technologies and related practices. Another significant point is that the effective method to modulate the metabolic level proposed in this work may hold considerable promise for other microbes used in food, medicine, and beverage industries.

Based on our findings, it remains a challenge to distinguish the specificity of cytochromes/enzymes with which the CDs interact in the cell membrane, periplasm, and cytoplasm, or the precise impact of the CDs on the metabolism and extracellular electron transfer, though clear electricity generation enhancement has been shown. A comprehensive investigation on in situ interaction of the CDs with the critical cytochromes/enzymes and detailed gene expression would be appropriate to shed light on the intricacies of the underlying molecular biology aspects.

## Methods

### Materials and characterization

The chemicals used in this work were naphthalene (98.0%, Aladdin), sodium (98.0%, Aladdin), ethylene glycol dimethyl ether (99.5%, Aladdin), triethylamine (99.5%, Aladdin), ethylenediamine (99.5%, Aladdin), n-propylamine (98%, Aladdin), BCA agent (Sangon), 2,3,5-Triphenyltetrazolium chloride (TTC) (Sinopharm group chemical reagent co. LTD), and ATP agent (Beyotime). Deionized (DI) water with a resistivity of 18.2 MΩ cm was obtained from a Milli-Q Water Purification System and used in all the experiments. The size and composition of the samples were characterized by TEM (Tecnai G20, FEI Co.,USA), AFM (MultiMode-8, Bruker, USA), XPS (ESCALAB 250Xi, Thermo Fisher Scientific, USA, using an Al Kα X-ray source), and FTIR (Nicolet 6700, Thermo Scientific, USA). The PL emission properties of the sample were evaluated by a fluorescence spectrophotometer (Fluoro max-4, Horiba, Japan). AFM analysis on the dehydrated cells was carried out by BioScan/Dimension Icon (Bruker, USA) and JPK Nanowizard IV (JPK, Germany) in force controlled tapping modes to ensure high resolution imaging without damaging the samples, using SNL-10 and MLCT (Bruker, USA) probes. The cross-section slice [fixed by glutaraldehyde (5%) and osmic acid (1%) without staining with a thickness of 50–60 nm] of the cells was imaged by TEM (Hitachi H-7650, Japan).

### Cryo-electron tomography

The experiments were performed at the iNANO Cryo-EM Facility, Aarhus University. The pristine/treated cells were added to C-flat 2/2 200 mesh copper grids (Protochips) and plunge frozen using a Leica GP2 plunge freezer. The vitrified samples were loaded into a Titan Krios (Thermo Fischer Scientific, USA) cryo-transmission electron microscope equipped with image Cs corrector and a Gatan Quantum 967 Special energy filter with a K2 Summit direct electron detector. Data were collected in counting mode using SerialEM with a filter slit width of 30 eV, a total dose of approximately 110 e Å^−2^ and a magnification of 26,000×. Data were processed using IMOD^45^ using fiducialless alignment.

### Synthesis and purification of the CDs

Typically, naphthalene (1.0 g) and sodium (0.2 g) were added into a flask (100 mL) with ethylene glycol dimethyl ether (20 mL). The mixture was ultrasonicated for 1 h to form sodium-naphthalene solution. Subsequently, triethylamine (0.2 mL) was added into the solution and stirred continuously for 4 h. Afterwards, the product was evaporated by the rotary evaporation apparatus under low pressure at 30 °C, then re-dispersed and centrifuged at 3821×*g*. The resultant supernatant was dialyzed by a dialysis membrane with a molecular weight cut off 1 KD for three days. The CD-X1 and CD-X2 samples were synthesized and purified using the same method, just using ethylenediamine (0.2 g) and n-propylamine (0.2 mL) to replace triethylamine.

### Growth of *S. oneidensis* MR-1 and its ΔOmcA/MtrC mutant

Wild type of *S. oneidensis* MR-1 (ATCC number of 700550TM) were used in this work. *S. oneidensis* MR-1 and ΔOmcA/MtrC mutant were cultured at 30 °C in LB broth (100 mL) aerobically at 150 rpm for 12 h until the logarithmic stationary phase was achieved.

### Biomass measurements

The biomass in the anode of *MEC-CD* and MEC-Control was determined by the protein concentration measured by BCA assay kit^46^.

### ETSA assay

The ETSA was determined using TTC as the artificial electron acceptor and acetone as extractant^47^. *S. oneidensis* MR-1 with/without the CDs were cultured under 30 °C for 12 h (*n* = 4). The mixture of the bacterial solution (5 mL), Tris-HCl buffer (1 mL), Na_2_SO_3_ (0.36%, 0.5 mL) and TTC (0.4%, 1 mL) was kept in a rotary incubator at 37 °C for 30 min, followed by addition of methanal (37%, 1 mL). After centrifuged under 2653×*g* for 5 min with the supernatant removed, acetone (5 mL) was added into the residue. The mixture was then incubated at 37 °C for 10 min, and centrifuged under 2653×*g* for 5 min. OD_485_ of supernatant was measured by ultraviolet–visible absorption spectroscopy. Standard curve was made by the same method using Na_2_S_2_O_4_ and TTC solution. The protein concentration was measured by BCA assay kit^46^. The calculation of ETSA follows Eq. (1):

1 $${\mathrm{ETSA}} = \frac{{{{D}}_{{\mathrm{485}}}{{V}}}}{{{{kwt}}}},$$

where *D*_485_ is the optical absorbance of the sample, *V* is the volume of acetone (5 mL), *k* is the slope of the standard curve, *w* is the protein weight, and *t* is the cell incubation duration (12 h).

### ATP assay

ATP levels were measured using a firefly luciferase based ATP assay kit^46^. *S. oneidensis* MR-1 with/without the CDs were cultured in MEC under 30 °C for 12 h (*n* = 4). ATP detection lysis buffer (0.5 mL) was added into the bacterial solution (0.5 mL) at 4 °C. The solution was centrifuged at 16,099 × *g* for 5 min (4 °C). ATP detection working dilution (100 μL) was mixed with supernatant (100 μL). Luminance was measured immediately using a monochromator microplate reader (BioTek). Standard curve was made by the same method using ATP standard liquid instead of bacterial solution. The protein concentration of the solution was measured by BCA assay kit.

### Electrochemical measurements

CV analysis was conducted by an electrochemical workstation (Metrohm, Autolab, B.V.) with Ag/AgCl and Pt wire as the reference and counter electrode, respectively. The CV test for *S. oneidensis* MR-1 and its ΔOmcA/MtrC mutant with a scan rate of 10 mV s^−1^ were performed within the potential region from –0.60 to 0.20 V and –0.60 to 0.40 V, respectively.

### MEC measurements

All the test and incubation of bacteria were carried out at 30 °C. Single chamber electrochemical reactor (42 mL) with 3-electrode configuration was used^5^. The electrolyte contained PBS (50 mmol L^−1^) with NH_4_Cl (6 mmol L^−1^), KCl (2 mmol L^−1^), and sodium lactate (18 mmol L^−1^). The reference and counter electrode were saturated Ag/AgCl electrode (0.198 V vs. standard hydrogen electrode) and Pt wire with a diameter of 0.1 mm, respectively. The working electrode was the carbon cloth (WOS 1002) with an area of 1 × 2 cm^2^ connected to the circuit by Ti wire. The electrochemical cells were connected to an eight-channel potentiostat (CHI600E, China) monitored by the computer. *S. oneidensis* MR-1 fed by the CDs (100 µg mL^−1^) were added into the electrochemical reactor to test the MECs performance. The MECs test of ΔOmcA/MtrC mutant cells followed a similar method. In this case, the concentration of sodium lactate was 5 mmol L^−1^, and the reactor volume was 50 mL.

### MFC measurements

Dual-chamber MFCs (100 mL) with the electrodes connected via a 1 kΩ external resistor was used to record voltage output of every 10 min using a data acquisition system (USB2801, ATD Co., China). Carbon cloth with a specific surface area of 1 × 2 cm^2^ and a proton exchange membrane were used as the electrode and the separator, respectively. Prior to the experiments, an anaerobic atmosphere was achieved. The catholyte was potassium ferricyanide (50 mmol L^−1^) in PBS (200 mmol L^−1^) at pH 7.0. The anolyte was PBS (200 mmol mL^−1^) at pH 7.0 with *S. oneidensis* MR-1 in LB solution (2 mL). The power density output curves were calculated by multiplying the constant voltage and its corresponding current.

### Statistical analysis

The experimental data were analyzed by two-tailed Student’s *t*-test. *p ≤ 0.05, **p ≤ 0.01, and ***p ≤ 0.001 were considered to be statistically significant.

### Reporting summary

Further information on research design is available in the Nature Research Reporting Summary linked to this article.

## Supplementary information

Supplementary Information

Reporting Summary

## Data Availability

The authors declare that all data supporting the findings of this study are available within the paper [and its supplementary information files]. Correspondence and requests for materials should be addressed to M.Y. or F.Z. or Y.S. or Y.F. or F.B.
