# Supplementary material for: Carbon dots-fed Shewanella oneidensis MR-1 for bioelectricity enhancement
Source: Nat Commun. 2020 Mar 13;11:1379. doi: 10.1038/s41467-020-14866-0 (PMC7070098; doi:10.1038/s41467-020-14866-0)
Supplement: Supplementary file 1 — Supplementary Information [file 41467_2020_14866_MOESM1_ESM.pdf]

Supplementary Information

Carbon Dots-Fed *Shewanella oneidensis* MR-1 for Bioelectricity Enhancement

Yang *et al.*

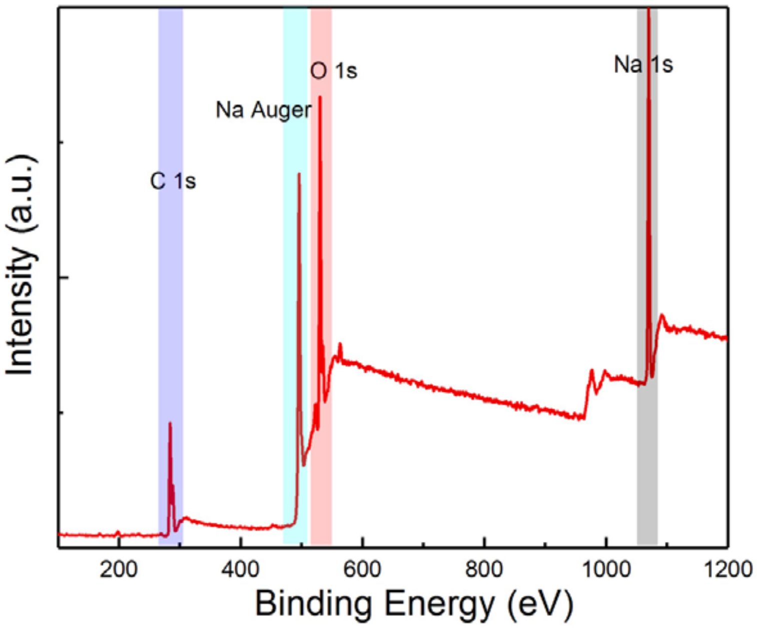

Supplementary Fig. 1. XPS survey of the CDs.

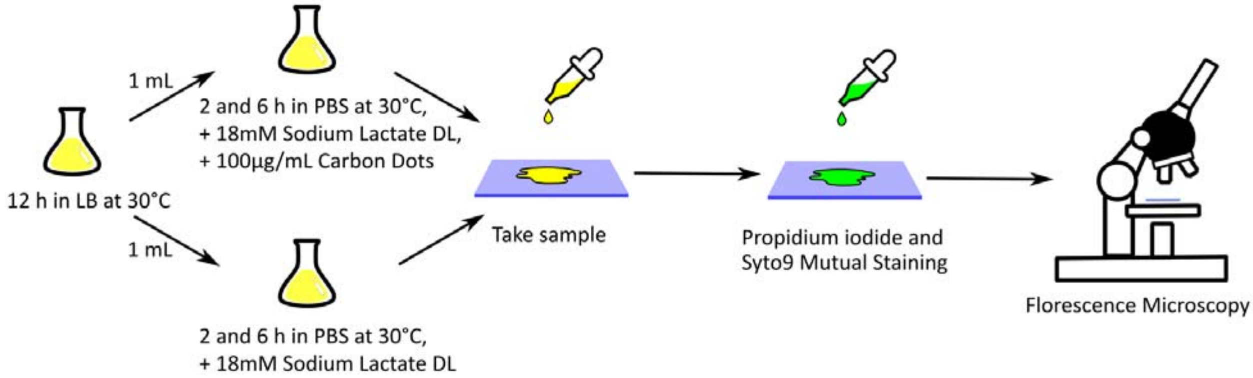

Supplementary Fig. 2. Protocol of staining *S. oneidensis* MR-1 using PI and Syto 9.

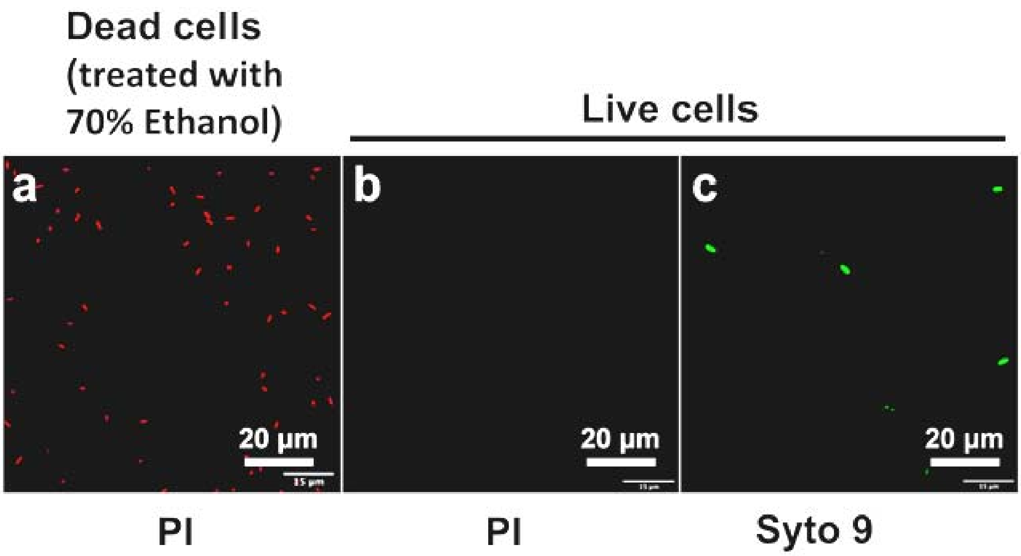

Supplementary Fig. 3. Fluorescence images of **a,** dead cells, and living cells stained by **b,** PI and **c,** Syto 9. Staining of dead cells by PI shows red color. Staining of living cells by PI shows no fluorescence signal. Staining of living cells by Styo 9 shows green color.

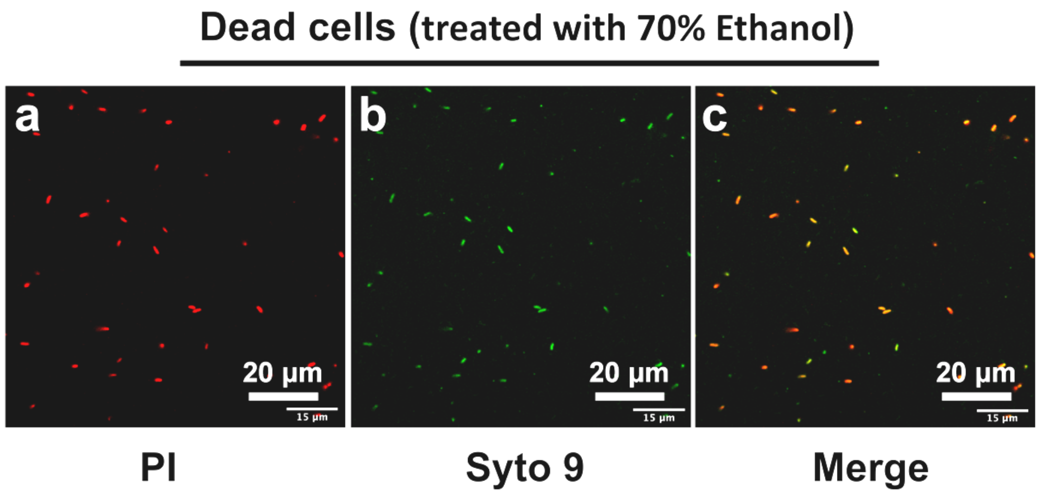

Supplementary Fig. 4. Fluorescence images of dead cells stained by **a,** PI, **b,** Syto 9, and **c,** the merged image of a and b.

According to the statistical analysis on the TEM images constructed using cryo-electron tomography, the size distribution of the dark spots in the pristine and CDs-fed *S. oneidensis* MR-1 cell was provided in Supplementary Figs. 2b–c, showing a spot size of 3.2 ± 0.5 nm in the pristine cell and additional larger spots (5.4 ± 0.8 nm) in the CDs-fed cells (Supplementary Fig. 2d).

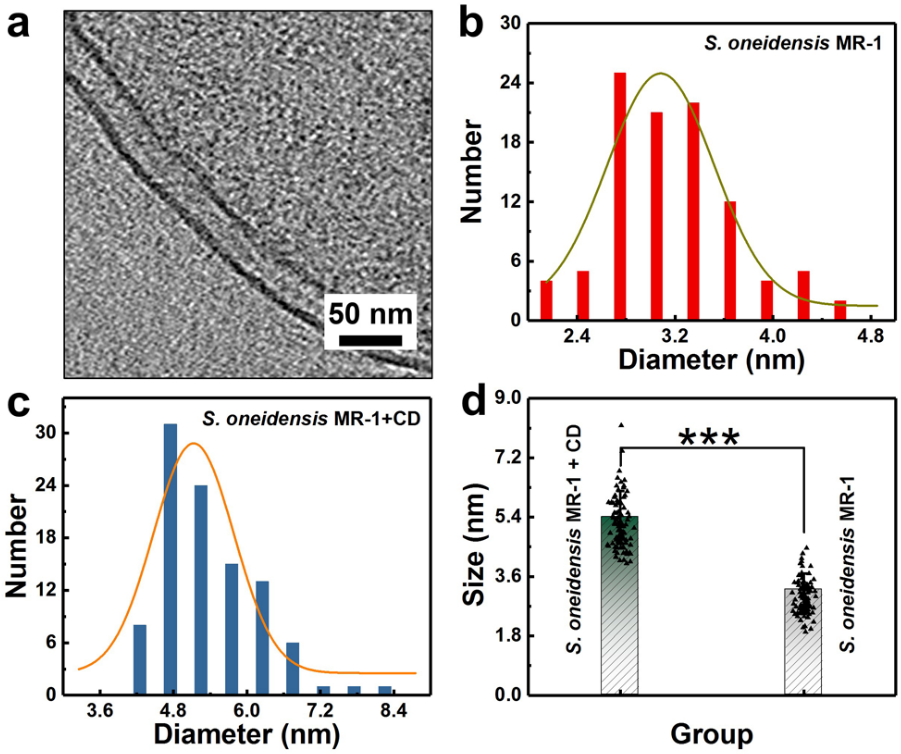

Supplementary Fig. 5. a, TEM image of a single pristine S. oneidensis MR-1 using cryo-electron tomography. Size distribution of **b,** the black spots in the pristine cell and **c,** the additional larger black spots in the CDs-fed cell, and **d**, the average spot size in the two cases (n = 100), based on the TEM images constructed using cryo-electron tomography. Data in panel d are expressed as the mean ± standard deviation. Two-tailed Student's t-test: *p*-value = 6.1×10^{-41}.

Based on the TEM images of cross-section slices of the CDs-fed cells, the size of the dark clusters in the CDs-fed *S. oneidensis* MR-1 was in the range of 20–50 nm (Supplementary Fig. 3b).

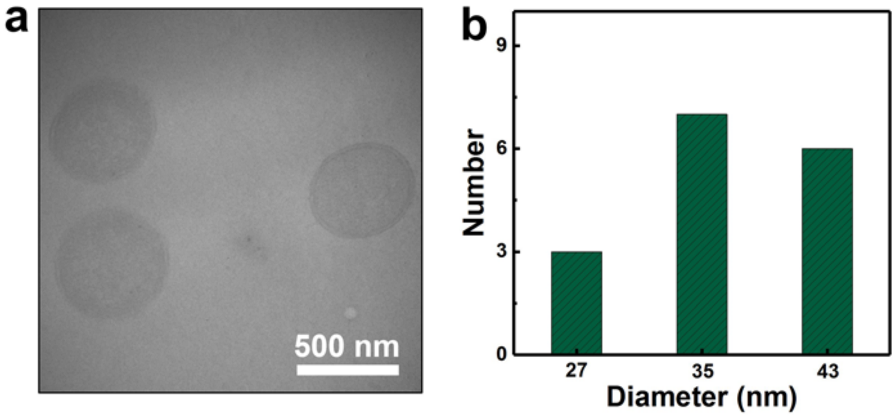

Supplementary Fig. 6. a, TEM image of cross-section slice of pristine *S. oneidensis* MR-1, showing uniform contrast without obvious black spots/clusters. **b,** Size distribution of the clusters in the CDs-fed cells based on the TEM images of their cross-section slices.

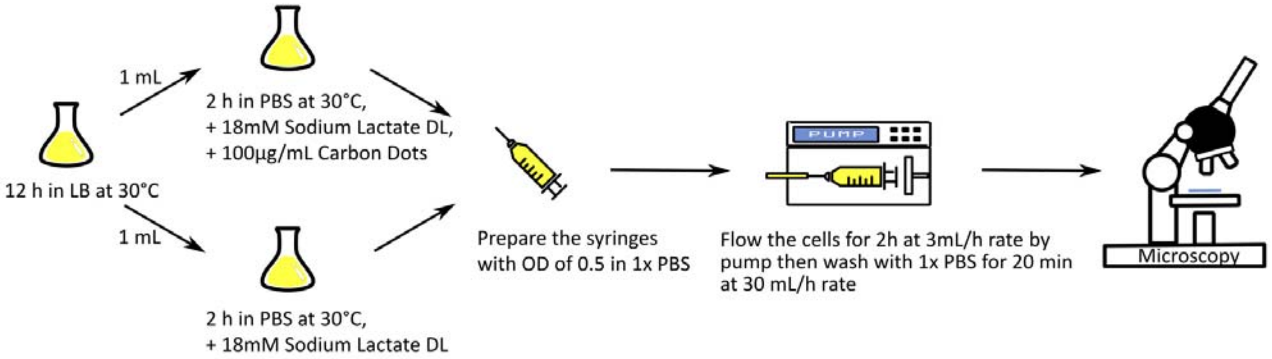

Supplementary Fig. 7. Protocol of flow-cell experiments of *S. oneidensis* MR-1 with/without the CDs' addition.

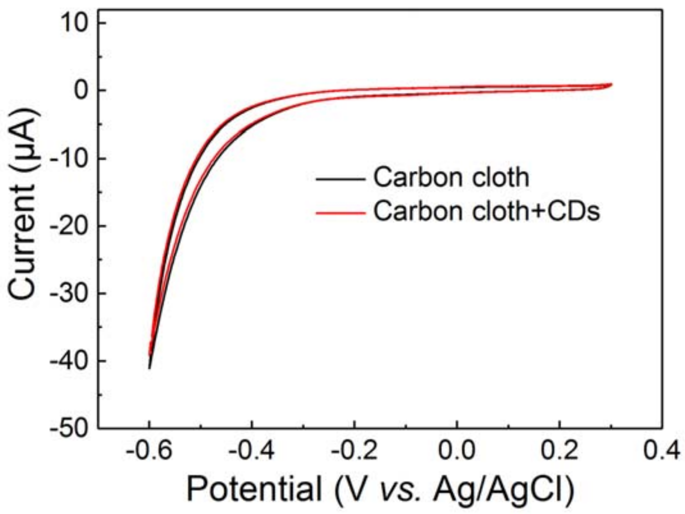

Supplementary Fig. 8. CV curves of carbon cloth alone and carbon cloth with the CDs.

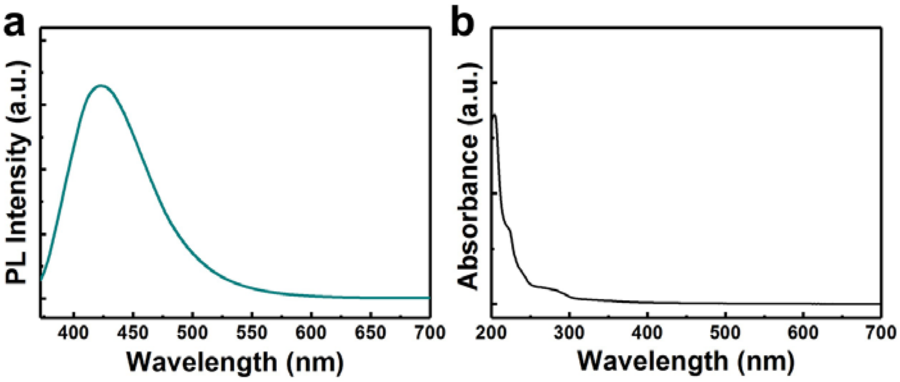

Supplementary Fig. 9. a, Photoluminescence (Excitation wavelength of 360 nm) and **b,** ultraviolet–visible absorption spectra of the CDs.

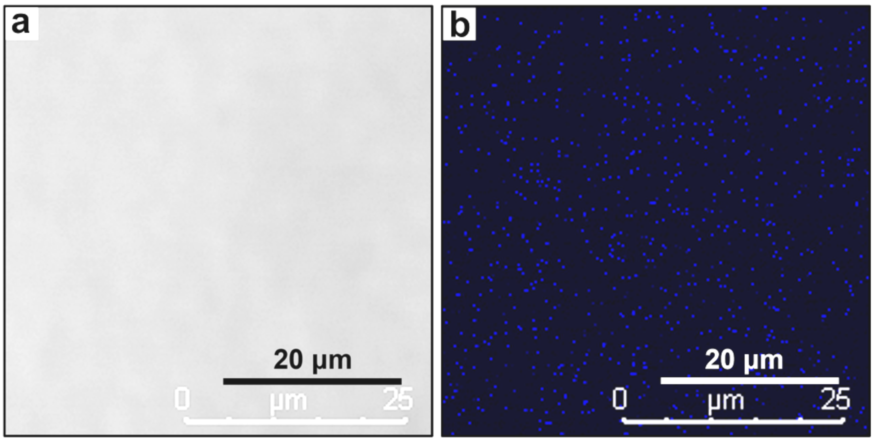

Supplementary Fig. 10. a, Bright-field and **b,** fluorescence images of CDs solution.

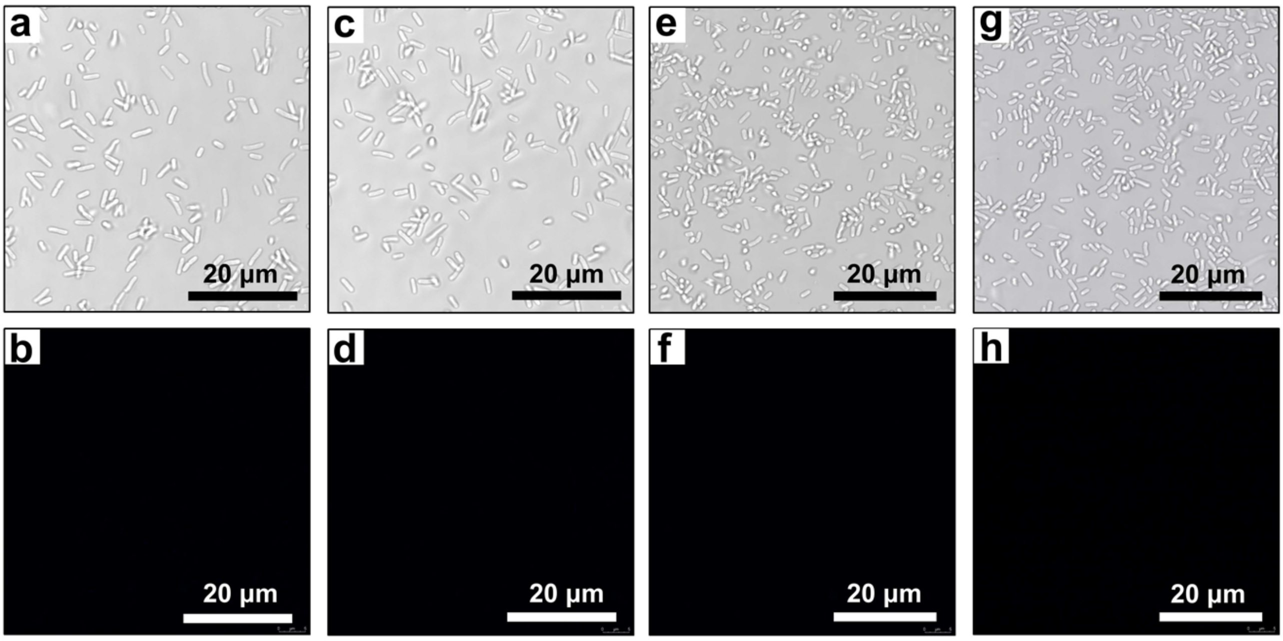

Supplementary Fig. 11. Bright-field and fluorescence images of *S. oneidensis MR-1* **a—b**, without and **c—d**, with the CDs' addition. Bright-field and fluorescence images of ∆OmcA/MtrC mutant cells **e—f**, without and **g—h**, with the CDs' addition.

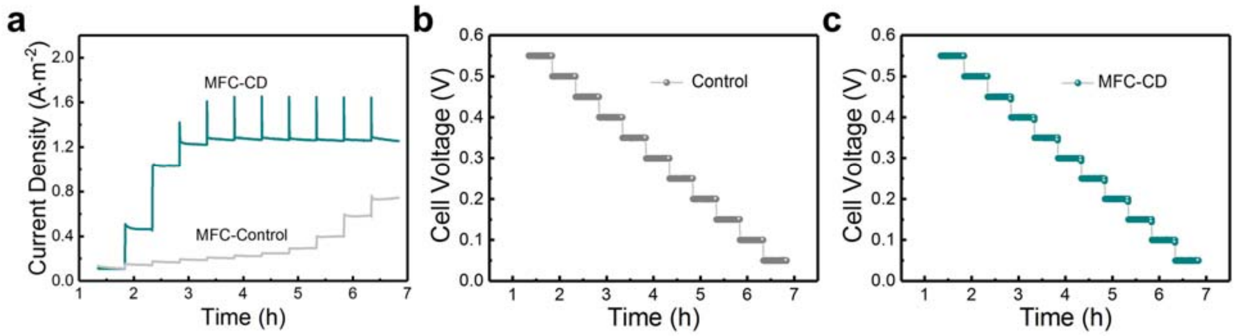

Supplementary Fig. 12. a, Current density of the *MFC-CD* and Control group. Constant cell voltage of **b**, the Control group and **c,** *MFC-CD* group.

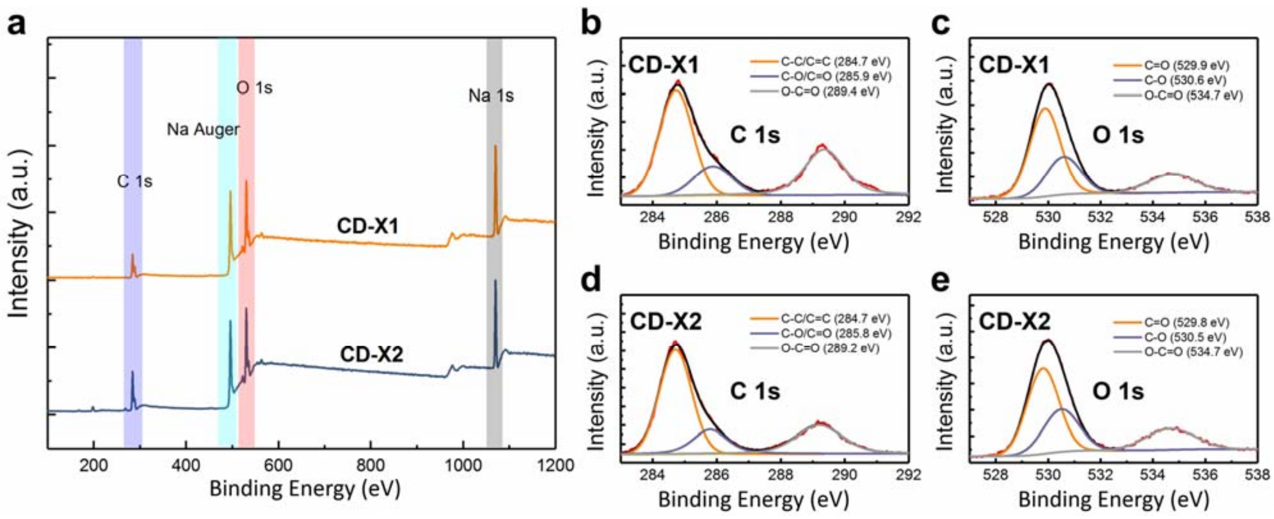

Supplementary Fig. 13. a, XPS survey of the CD-X1 and CD-X2. **b,** C *1s* and **c,** O *1s* XP spectra of the CD-X1. **d,** C *1s* and **e,** O *1s* XP spectra of the CD-X2.

Supplementary Table 1. Content analysis based on C *1s* XP spectra of the CD, CD-X1 and CD-X2.

	C-C/C=C (%)	C-OH/C=O (%)	-O-C=O (%)
CD	47	19	34
CD-X1	52	16	32
CD-X2	57	17	26

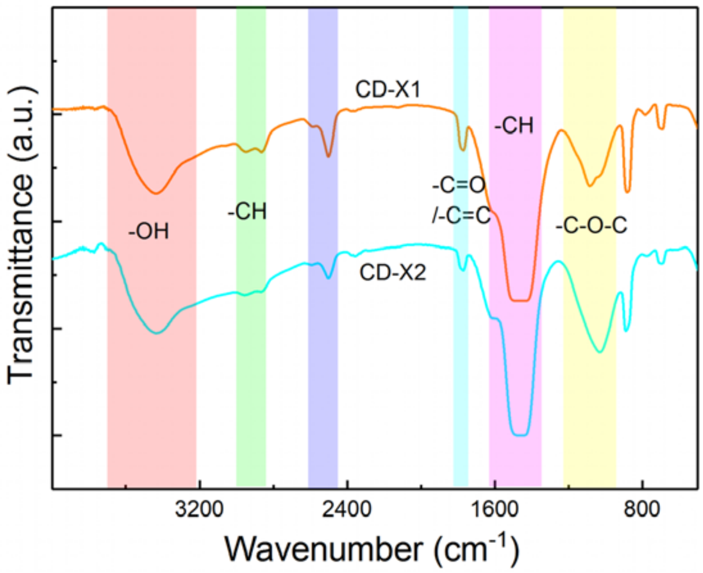

Supplementary Fig. 14. FTIR spectra of the CD-X1 and CD-X2.

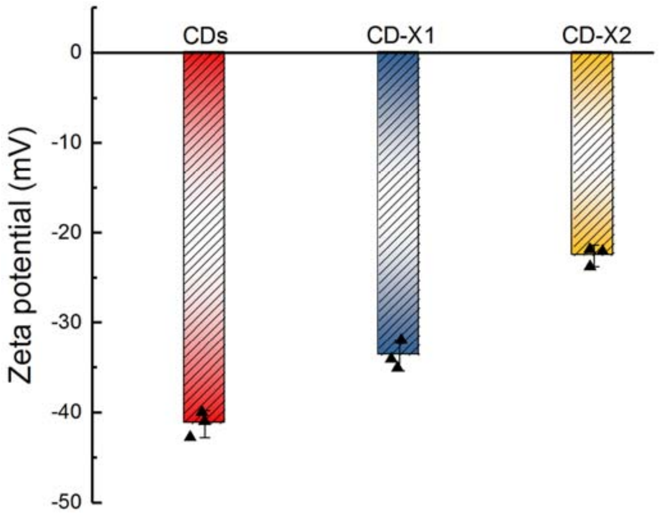

Supplementary Fig. 15. Zeta potential of the CD, CD-X1 and CD-X2. Data are expressed as the mean ± standard deviation.

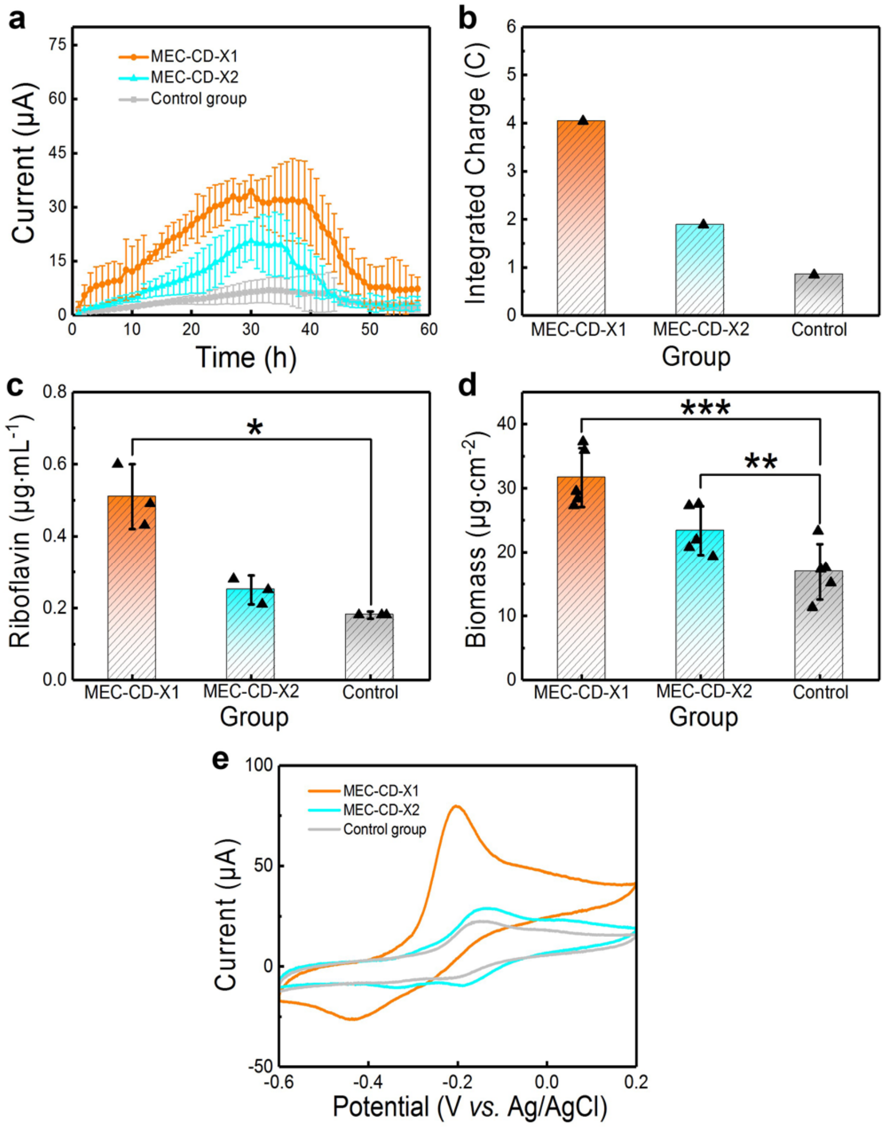

Supplementary Fig. 16. a, Current output (*n* = 3), **b,** Integrated charge, **c,** Riboflavin concentration (*n* = 3), **d,** Biomass (*n* = 5) and **e,** CV curves of the *MEC-CD-X1, MEC-CD-X2* and Control group, where *MEC-CD-X1 and MEC-CD-X2* represent the MEC of Wide Type *S. oneidensis* MR-1 with CD-X1 and CD-X2, respectively, and Control group represents the MEC of Wide Type *S. oneidensis* MR-1. Data in panel a, c and d are expressed as the mean ± standard deviation. Two-tailed Student's *t*-test: *$p < 0.05$, **$p < 0.01$ and ***$p < 0.001$.
